# Bimolecular Complementation to Visualize Filovirus VP40-Host Complexes in Live Mammalian Cells: Toward the Identification of Budding Inhibitors

**DOI:** 10.1155/2011/341816

**Published:** 2011-10-18

**Authors:** Yuliang Liu, Michael S. Lee, Mark A. Olson, Ronald N. Harty

**Affiliations:** ^1^Department of Pathobiology, School of Veterinary Medicine, University of Pennsylvania, 3800 Spruce Street, Philadelphia, PA 19104, USA; ^2^Department of Cell Biology and Biochemistry, U. S. Army Medical Research Institute of Infectious Diseases, 1425 Porter Street, Frederick, MD 21702, USA

## Abstract

Virus-host interactions play key roles in promoting efficient egress of many RNA viruses, including Ebola virus (EBOV or “e”) and *Marburg virus* (MARV or “m”). Late- (L-) domains conserved in viral matrix proteins recruit specific host proteins, such as Tsg101 and Nedd4, to facilitate the budding process. These interactions serve as attractive targets for the development of broad-spectrum budding inhibitors. A major gap still exists in our understanding of the mechanism of filovirus budding due to the difficulty in detecting virus-host complexes and mapping their trafficking patterns in the natural environment of the cell. To address this gap, we used a bimolecular complementation (BiMC) approach to detect, localize, and follow the trafficking patterns of eVP40-Tsg101 complexes in live mammalian cells. In addition, we used the BiMC approach along with a VLP budding assay to test small molecule inhibitors identified by *in silico* screening for their ability to block eVP40 PTAP-mediated interactions with Tsg101 and subsequent budding of eVP40 VLPs. We demonstrated the potential broad spectrum activity of a lead candidate inhibitor by demonstrating its ability to block PTAP-dependent binding of HIV-1 Gag to Tsg101 and subsequent egress of HIV-1 Gag VLPs.

## 1. Introduction

Filoviruses are human pathogens that cause severe hemorrhagic disease and are potential agents of bioterrorism [1, 2]. EBOV and MARV are BSL-4 agents and NIAID Category A priority pathogens due to their association with high fatality rates and lack of approved vaccines or antivirals [2]. Filoviruses are enveloped, nonsegmented, negative-strand RNA viruses with an approximately 19.0-kilobase genome encoding the nucleoprotein (NP), VP35, matrix protein (VP40), attachment glycoprotein (GP), VP30, VP24, and RNA polymerase protein (L) [3]. VP40 is the major component of virions, and expression of VP40 alone in mammalian cells is sufficient to generate extracellular virus-like particles (VLPs), which resemble authentic virions in overall morphology [4–10]. Late- (L-) domain motifs conserved in the VP40 proteins are critical for efficient egress of VLPs and virions, as they function by hijacking specific host proteins involved in vacuolar protein sorting (vps) pathways to facilitate the final step of virus-cell separation [3, 6, 10–14]. EBOV VP40 (eVP40) possesses two L-domain motifs (PTAP and PPEY) at its N-terminus (_7_-PTAPPEY-_13_) [4, 6] whereas MARV VP40 (mVP40) and NP (mNP) contain single PPPY and PTAP L-domain motifs, respectively [12, 15]. Various approaches such as protein affinity chromatography, GST-pulldowns, and yeast two-hybrid screens have been used successfully to detect these functionally relevant L-domain mediated virus-host interactions *in vitro* [6, 12, 15]. For example, the PTAP L-domain of eVP40 recruits host Tsg101, a component of the cellular ESCRT (endosomal sorting complex required for transport) pathway involved in sorting monoubiquitinated proteins into multivesicular bodies (MVBs) [3, 6, 10, 12, 15–22] whereas the PPEY motif of eVP40 mediates an interaction with host Nedd4 ubiquitin ligase [4] leading to ubiquitination of eVP40 and enhanced VLP egress [4, 10, 19, 23, 24]. Despite these *in vitro* studies, detection and visualization of these virus-host complexes, as well as the intracellular trafficking patterns of these complexes in the natural environment of the host cell remain elusive. 

 To address these gaps and to identify effective small molecule inhibitors of filovirus budding, we used a bimolecular complementation (BiMC) assay [25–28] with *Venus* enhanced yellow fluorescence protein (EYFP) to investigate filovirus VP40-host interactions in mammalian cells in real time [8]. *Venus* EYFP, a GFP variant containing a novel mutation of F46L, can be split into N- and C-terminal fragments, and reconstitution of these two EYFP fragments mediated by a protein-protein interaction results in an essentially irreversible fluorescent signal. This approach is useful for detecting and recording transient interaction events, allowing for detection of short-lived and/or weakly-associated protein-protein interactions in intact living cells [26, 28–31]. Using this approach, we were able not only to visualize an eVP40-Tsg101 interaction in live mammalian cells [8], but also were able to localize and follow the migration of eVP40-Tsg101 complexes in live cells. Lastly, we used BiMC and VLP budding assays to assess the specific inhibitory effects of small molecule compounds designed to block PTAP-mediated virus-host interactions and subsequent virus budding. 

## 2. Materials and Methods

### 2.1. Cells, Plasmids, and Antisera

 Human 293T cells were maintained in DMEM enriched with 10% FBS. All chimeric constructs were cloned into the pCAGGS expression vector. Plasmids eVP40-WT and eVP40-ΔPT/PY have been described previously [6]. The original MARV VP40 expression plasmid was kindly provided by Stephan Becker (Marburg, Germany). Plasmid pCS2 containing full-length *Venus* EYFP was generously provided by Roselyn J. Eisenberg, Gary Cohen, and Doina Atanasiu (University of Pennsylvania School of Dental Medicine). An HIV-1 Gag expression construct and anti-Gag antiserum were kindly provided by Paul Bates (University of Pennsylvania). NYFP and CYFP fragments were PCR-amplified and fused independently with full-length Tsg101, eVP40, mVP40, HIV Gag, or L-domain mutants of eVP40 or Gag in pCAGGS by standard cloning techniques [8]. Plasmid CYFP-mVP40 contains an in-frame FLAG epitope tag between the CYFP fragment and mVP40 ORF. Anti-eVP40 monoclonal antiserum was kindly provided by Dr. Gene Olinger (USAMRIID, Ft. Detrick, MD) [6]. Mouse monoclonal antibody against the FLAG epitope (Sigma-Aldrich) was used according to the manufacturer's instructions. Goat polyclonal antibody against pericentrin-B was purchased from Santa Cruz Biotechnology. Small-molecule compound 5539-0062 (ChemDiv, San Diego, CA) was dissolved in dimethyl sulfoxide (DMSO, FisherBiotech) at a concentration of 10 mM and stored at −20°C.

### 2.2. VLP Budding Assays

 Human 293T cells were singly or co-transfected with the indicated plasmids using Lipofectamine (Invitrogen) in Opti-MEM (Invitrogen) according to the manufacturer's directions. VLP budding assays and western blotting were performed as described previously [8]. 

### 2.3. BiMC, Immunofluorescence, and Confocal Microscopy

 Human 293T cells were grown on glass coverslips in six-well plates and cotransfected with the indicated plasmids. At 24 hours after transfection, cells were washed with PBS, fixed with cold methanol/acetone (vol/vol, 1 : 1) and stained with appropriate primary and second antibodies as indicated. Cells were washed as described above and subsequently stained with 4′,6′-diamidino-2-phenylindole (DAPI) for 10 min at room temperature. Cells were washed four times with PBS, and affixed to glass slides with Prolong Antifade (Invitrogen/Molecular Probes). Slides were viewed using an LSM-510 Meta confocal microscope (Carl Zeiss). For live cell imaging, 293T cells were seeded in glass bottom microwell dishes and transfected with the indicated plasmids. At 4 h after transfection, YFP fluorescence was observed using spinning-disk confocal microscopy in the presence of 5% CO_2_ and humidity at 37°C. Cells were monitored for YFP fluorescence for a period of 20–24 hours after transfection. 

### 2.4. siRNA Transfection

Tsg101-specific siRNA and random siRNA (used as a negative control) were purchased from Dharmacon Inc. Transfection was performed using Lipofectamine 2000 (Invitrogen) according to the manufacturer's instructions. Briefly, 293T cells were seeded in 24-well plates for 2 hours. Cells were then transfected with 20 nM of Tsg101-specific or random siRNA. At 24 h.p.t. the cells were transfected a second time with identical amounts of siRNA and the indicated plasmid(s). At 48 h.p.t. the cells were washed, fixed, and YFP fluorescence was detected as described above. Cells extracts were harvested in RIPA buffer and used in western blotting assays.

### 2.5. Small Molecule Inhibitors of VP40 Budding

Human 293T cells seeded in 6-well plates were pretreated with either vehicle (DMSO) alone, or compound 5539-0062 in DMSO at the indicated concentrations for 1 h at 37°C. Cells were subsequently transfected with the indicated plasmids. Viral proteins in cell extracts and VLPs were detected by western blotting as described above.

## 3. Results

### 3.1. Generation of EYFP Fusion Plasmids and Expression of EYFP Fusion Proteins

**Figure 1:**
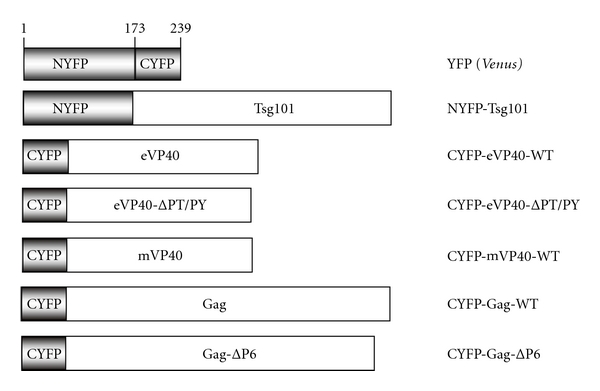
Schematic diagram of EYFP fusion proteins. The N-terminal half of EYFP (aa 1–173) was joined in-frame to full-length Tsg101, and the C-terminal half of EYFP (aa 174–239) was joined in-frame to the indicated WT or L-domain mutant VP40 or HIV-1 Gag proteins. A FLAG epitope tag is positioned at the N-terminus of mVP40-WT, and between the CYFP fragment and mVP40 in construct CYFP-mVP40-WT. The CYFP-Gag-ΔP6 construct was made by fusing the CYFP fragment in-frame with Gag L-domain deletion mutant (the entire p6 region containing L-domain motif PTAP is deleted).

We generated a series of plasmids (some of which have been described previously, see [8]) for use in the BiMC assay that contain either the N-terminal EYFP fragment (residues 1–173, denoted NYFP), or the C-terminal EYFP fragment (residues 174–239, denoted CYFP) joined in-frame to host Tsg101, or to WT and L-domain mutants of eVP40, mVP40, and HIV-1 p55-Gag (Figure 1). All plasmids were verified by automated DNA sequencing, and expression of all YFP fusion proteins was confirmed by western blotting [8]; (Liu and Harty, data not shown). Importantly, fusion of the CYFP fragment to the N-termini of all viral proteins did not affect protein expression, nor the well-characterized, L-domain-dependent budding properties of these viral matrix proteins (Liu and Harty, data not shown); [8, 32]. 

### 3.2. Detection, Localization, and Trafficking of eVP40-Host Interactions in Mammalian Cells Using BiMC

**Figure 2:**
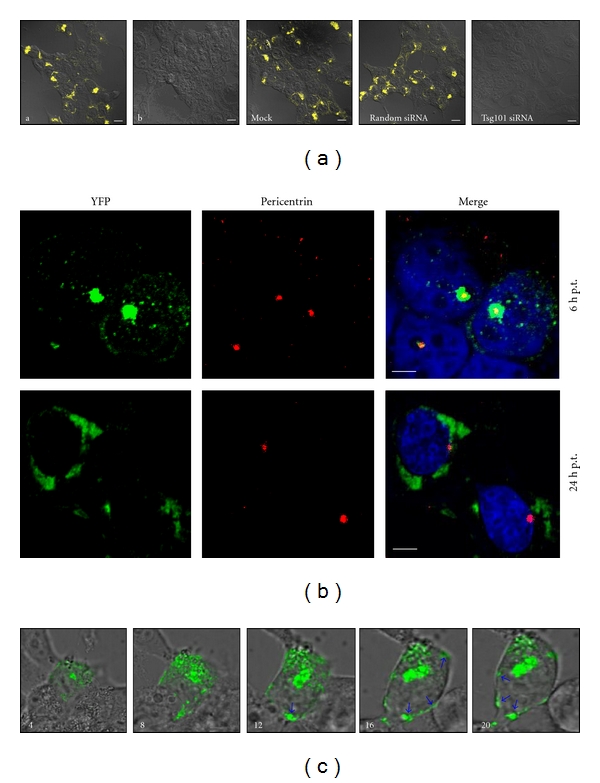
BiMC detecting eVP40-Tsg101 interactions and trafficking of Tsg101/eVP40 complex. (a) Human 293T cells grown on coverslips in 6-well plates were transfected with NYFP-Tsg101 + CYFP-eVP40-WT (a) or NYFP-Tsg101 + CYFP-eVP40-ΔPT/PY. (b) Cells were washed, fixed, and examined for YFP fluorescence by confocal microscopy at 24 hours p.t. Cells were mock-transfected, or transfected with 20 nM of Tsg101-specific or random siRNA. Twenty four hours later, cells were mock-transfected or transfected with the same amount of siRNA along with NYFP-Tsg101 + CYFP-eVP40. After an additional 24 hours, cells were examined for YFP fluorescence as described above. (b) Colocalization eVP40-Tsg101 complexes with pericentrin. BiMC assay showing colocalization between pericentrin-B and YFP fluorescence from 293T cells expressing NYFP-Tsg101 + CYFP-eVP40-WT at 6 hours p.t. (top panel) and 24 hours p.t. (bottom panel). (c) DIC images of a single 293T cell co-expressing NYFP-Tsg101 + CYFP-eVP40-WT at 4, 8, 12, 16, and 20 hours p.t. Blue arrows indicate Tsg101/eVP40 complex accumulation at the plasma membrane.

 Recently, we reported that the BiMC approach could be used to detect viral protein-protein interactions in live mammalian cells [32]. Here, we used a similar approach to detect and map the intracellular localization of these filovirus-host complexes, and follow these complexes in real time as they traffic through the cell. Briefly, human 293T cells were cotransfected with plasmids expressing NYFP-Tsg101 + CYFP-eVP40-WT, or NYFP-Tsg101 + CYFP-eVP40-ΔPT/PY (L-domain deletion mutant of eVP40), and cells were examined for YFP fluorescence at 24 h.p.t. No background fluorescence was detected when fusion plasmids were transfected singly ([32], Liu et al., data not shown). YFP fluorescence was observed readily and reproducibly in 293T cells coexpressing NYFP-Tsg101 + CYFP-eVP40-WT (Figure 2(a), panel a); however, this signal was virtually absent in cells coexpressing NYFP-Tsg101 + CYFP-eVP40-ΔPT/PY (Figure 2(a), panel b) [32]. Similar results were obtained in cell lines other than human 293T cells including, Huh7, HeLa, and A549 cells (Liu and Harty, data not shown). The use of the L-domain deletion mutant of eVP40 not only validates the specificity of the observed EYFP signal (Figure 2(a), panel a), but also confirms the overall feasibility of using the BiMC approach to detect and record these transient virus-host interactions in mammalian cells [32]. It should be noted that the CYFP-eVP40-ΔPT/PY protein was shown to be functional in the BiMC assay, as it interacted with NYFP-eVP40-WT to form eVP40-WT/eVP40-ΔPT/PY complexes which resulted in YFP fluorescent cells [32]. 

 To further prove that the observed YFP fluorescence (Figure 2(a), panel a) was due to a specific interaction between eVP40 and Tsg101, we cotransfected cells with random or Tsg101-specifc siRNAs. Briefly, human 293T cells grown on coverslips in 6-well plates were mock-transfected, or transfected with 20 nM of Tsg101-specific or random siRNA. Twenty-four hours later, cells were mock-transfected, or transfected with the same amount of siRNAs along with NYFP-Tsg101 + CYFP-eVP40 plasmids (Figure 2(a)). After an additional 24 hours, cells were examined by confocal microscopy for YFP fluorescence. A strong YFP signal was observed in cells expressing Tsg101/eVP40-WT in the presence of no siRNA (mock), or random siRNA (Figure 2(a)). In contrast, YFP fluorescence was virtually absent in cells receiving Tsg101-specific siRNA (Figure 2(a)). Importantly, the concentration of Tsg101-specfic siRNAs used above was shown by western blot to knockdown expression of Tsg101 in 293T cells by >90% (Liu and Harty, data not shown; [6]).

### 3.3. Trafficking and Intracellular Localization of Tsg101/eVP40 Complexes in Live Cells

Once the specificity of the NYFP-Tsg101 + CYFP-eVP40-WT interaction was confirmed, we sought to determine the kinetics of association and intracellular origin of this virus-host complex, and also follow its movement through the cell in real time. The YFP fluorescence generated at early times p.t. in cells co-expressing NYFP-Tsg101 + CYFP-eVP40-WT appeared to be punctate in nature and localized adjacent to the nucleus (Figure 2(b)). This intracellular position resembled that of the microtubule organizing center (MTOC). Therefore, we sought to determine whether the NYFP-Tsg101 + CYFP-eVP40-WT complex was initially forming at the MTOC by using an antibody to pericentrin-B: an MTOC marker protein (Figure 2(b)). Indeed, the Tsg101/eVP40-WT complex was first visible between 3–4 hours p.t., and this complex colocalized strongly with pericentrin-B at 6 hours p.t. (Figure 2(b), top row). In contrast, colocalization of the Tsg101/eVP40-WT complex with pericentrin-B was not evident at later times p.t. (12–24 hours, Figure 2(b), bottom row). These findings suggest that the Tsg101/eVP40 complex may traffic via the microtubule network to the eventual site of budding at the plasma membrane. It should be noted that additional cellular markers including calnexin (ER), Giantin (*cis*-Golgi), and EEA1 (early endosome) were used in similar colocalization experiments; however, the Tsg101/eVP40-WT complex did not exhibit any significant level of colocalization with these host proteins (Liu and Harty, data not shown). 

 Further evidence supporting the movement of the Tsg101/eVP40 complex from the MTOC to the plasma membrane was obtained by continuous monitoring of the YFP signal in co-transfected cells over a 16-hour period (Figure 2(c)). Real-time images of a single cell co-expressing NYFP-Tsg101 + CYFP-eVP40-WT at the indicated times p.t. illustrates the changes in intracellular localization of Tsg101-eVP40-WT complexes from their initial formation at the MTOC between 3–4 hours after transfection, to their enhanced accumulation at distinct patches on the plasma membrane (Figure 2(c)). Taken together, the properties exhibited by Tsg101-eVP40-WT complexes in the natural environment of the cell appear to represent functional recruitment of Tsg101 by eVP40 leading to efficient budding. 

### 3.4. Small Molecule Inhibitors of Filovirus VP40-Host Interactions and VP40 VLP Budding

**Figure 3:**
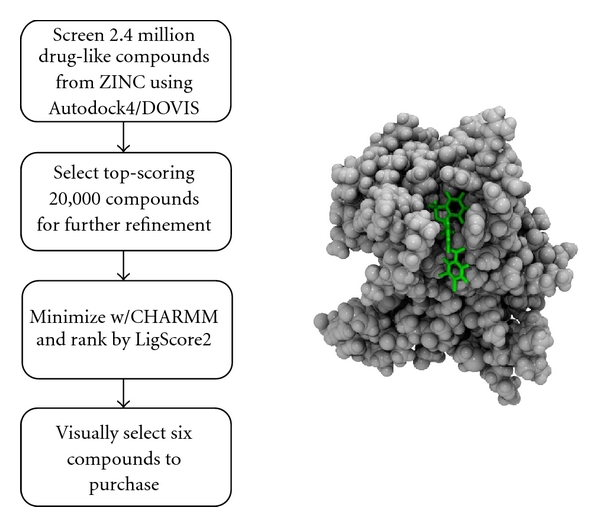
Strategy for computational screening of budding inhibitors. The stereo view of the PTAP motif (green) in its binding groove on the UEV domain of Tsg101.

Viral L-domains are attractive targets for host-oriented therapeutics which may possess broad-spectrum activity against a plethora of L-domain containing RNA viruses [33]. For example, a five amino acid cyclic peptide was reported recently to disrupt the interaction between Tsg101 and the PTAP L-domain motif of HIV-1 Gag leading to diminished egress of Gag VLPs [34, 35]. Our strategy to identify inhibitors of filovirus budding was to perform *in silico* screening of the ZINC drug-like library (2.4 million compounds) against the NMR-derived structure of human Tsg101 UEV domain (PDB id: 1M4P, conformer #1) focusing on the binding pocket of the PTAP peptide (Figure 3) [34, 36, 37]. The PTAP binding pocket is outlined roughly by residues Y63, Y68, I70, M95, F142, and S143 of Tsg101. High-throughput computational screening was performed with the AutoDock program in the DOVIS pipeline [38, 39]. The top 20,000 complexes from the initial screen were minimized in CHARMM with the MMFF force field and reranked using Accelrys Ligscore2 [40–42]. 

**Figure 4:**
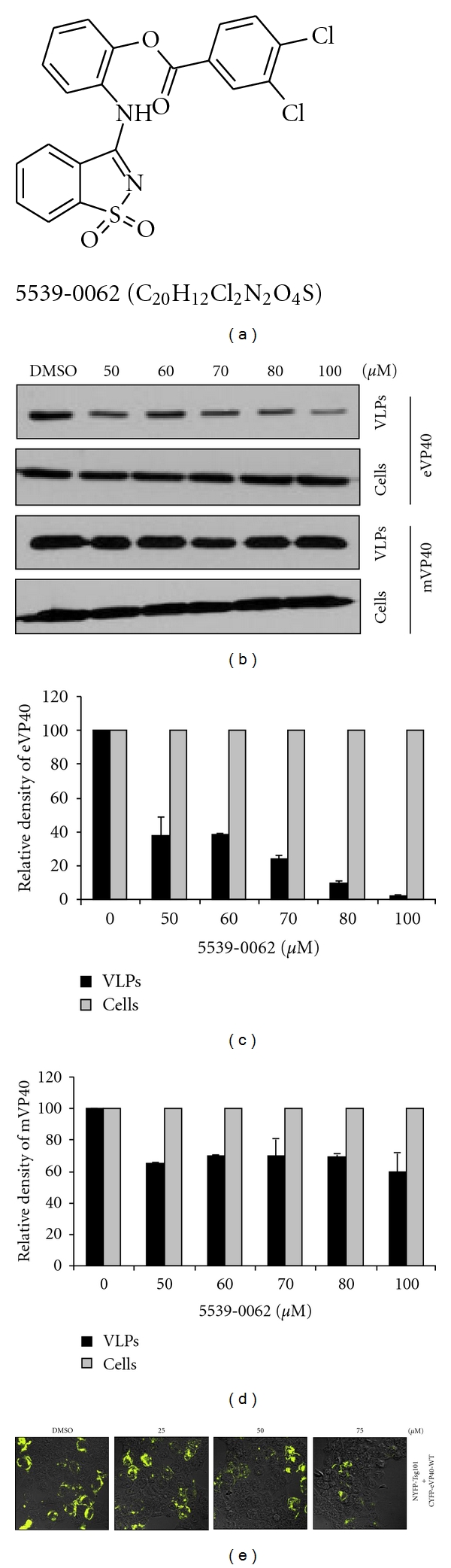
Inhibition of VP40-Tsg101 interactions and VLP budding. (a) Chemical structure of compound 5539-0062. (b) Budding assay and western blotting for eVP40 and mVP40 in VLPs and cell extracts from human 293T cells treated with DMSO alone, or the indicated concentrations of PTAP-inhibitor 5539-0062. (c) Bar graph of the relative amounts of eVP40-WT in cells and VLPs (part B) determined using Dot-Blot, LabWorks software (UVP). (d) Bar graph of the relative amounts of mVP40-WT in cells and VLPs (part B) determined using Dot-Blot, LabWorks software (UVP). (e) BiMC of human 293T cells expressing NYFP-Tsg101 + CYFP-eVP40-WT and treated with DMSO alone, or the indicated concentrations of PTAP-inhibitor 5539-0062. Cells were examined for YFP fluorescence at 24 hour p.t., and positive cells were quantified by FACS analysis. The average values were as follows: DMSO alone = 100%, 25 *μ*M = 84%, 50 *μ*M = 68%, and 75 *μ*M = 24%.

We tested the top six scoring compounds using both BiMC and VLP budding assays for their ability to disrupt either an eVP40-Tsg101, or mVP40-Tsg101 interaction and subsequent VLP egress. Of the six molecules tested, compound 5539-0062 (Figure 4(a)) exhibited PTAP-specific inhibition of eVP40 VLP egress and of eVP40-Tsg101 complex formation. Briefly, human 293T cells were first treated for 1 hour with either carrier (DMSO) alone, or increasing concentrations of compound 5539-0062, and then budding of eVP40 or mVP40 VLPs was evaluated and quantified (Figures 4(b)–4(d)). Compound 5539-0062 inhibited PTAP-dependent budding of eVP40-WT VLPs by >50% at lower concentrations, and by >90% at higher concentrations (Figures 4(b) and 4(c)). In contrast, PTAP-independent budding of mVP40-WT VLPs was reduced by <2-fold at all concentrations tested (Figures 4(b) and 4(d)). Expression controls for eVP40-WT and mVP40-WT in cells remained unaltered at all drug concentrations tested (Figures 4(b)–4(d)). It should be noted that concentrations of compound 5539-0062 used for the above experiments exhibited no significant toxicity in human 293T cells in culture as determined by XTT colorimetric assay (Liu and Harty, data not shown). Consistent with the findings of our VLP budding assays (Figures 4(b)–4(d)), compound 5539-0062 blocked the interaction between NYFP-Tsg101 + CYFP-eVP40-WT as determined by BiMC (Figure 4(e)) and quantitation of YFP fluorescing cells by FACS analysis. Thus, compound 5539-0062 was capable of specifically inhibiting PTAP-dependent budding of eVP40 VLPs by disrupting the eVP40-Tsg101 interaction while having no effect on PTAP-independent budding of mVP40 VLPs.

To further prove the PTAP-specific inhibitory effect of compound 5539-0062 and assess its ability to inhibit budding of infectious virus, we infected 293T cells with either VSV-WT (containing a PPxY-type L-domain), or VSV-M40; a VSV recombinant containing the PTAP-type L-domain from eVP40 in place of the normal VSV PPxY-type L-domain [18] (Table 1). In two independent experiments, cells were infected at an MOI of 1.0 for 8 hours, and virus released into the supernatant was harvested and titered on BHK-21 cells. Equivalent titers of VSV-WT and VSV-M40 were obtained in the presence of vehicle (DMSO) alone (Table 1). While the titers of VSV-WT did decrease slightly in the presence of increasing concentrations of 5539-0062, the titers of VSV-M40 were reproducibly reduced by 3–10-fold more than those of VSV-WT in the presence of increasing concentrations of compound 5539-0062 (Table 1). These data correlate well with those shown in Figure 4, and further support the specificity of compound 5539-0062 to inhibit viral PTAP L-domain function by inhibiting budding of an infectious VSV recombinant expressing the PTAP-type L-domain from eVP40.

### 3.5. Compound 5539-0062 Inhibits HIV-1 Gag-Tsg101 Interaction and PTAP-Dependent Gag VLP Budding

**Figure 5:**
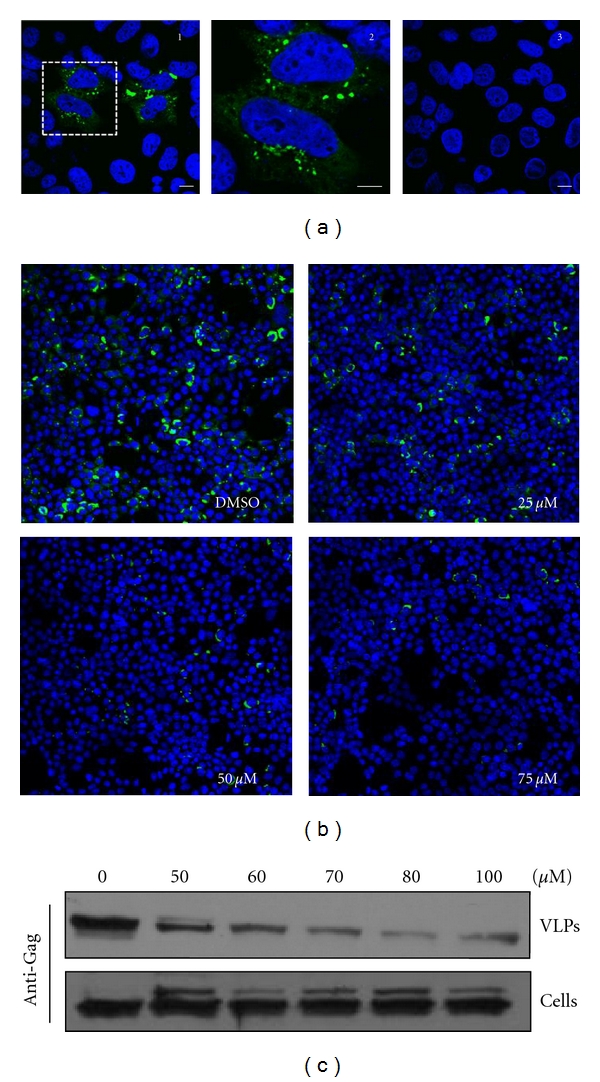
Inhibition of HIV-1 Gag-Tsg101 interaction and VLP budding. (a) Human 293T cells grown on coverslips in 6-well plates were transfected with NYFP-Tsg101 + CYFP-Gag-WT (panels 1 and 2) or NYFP-Tsg101 + CYFP-Gag-ΔP6 (panel 3). Cells were washed, fixed, and examined for YFP fluorescence by confocal microscopy at 24 hours p.t. The image within the dotted square in panel 1 was enlarged and shown panel 2. (b) Human 293T cells grown on coverslips in 6-well plates were transfected with NYFP-Tsg101 + CYFP-Gag-WT in the presence of DMSO alone, or 25, 50, or 75 *μ*M of compound 5539-0062 as indicated. Cells were washed, fixed, and examined for YFP fluorescence by confocal microscopy at 24 hours p.t. (c) Standard budding assay and western blotting for Gag in VLPs and cell extracts from human 293T cells treated with DMSO alone, or the indicated concentrations of PTAP-inhibitor 5539-0062.

The p6 region of HIV-1 Gag contains a well-defined PTAP L-domain motif that mediates efficient budding of HIV-1 virions and Gag VLPs by recruiting Tsg101 in a manner similar to that used by eVP40 [16, 17, 20]. To determine whether PTAP inhibitor 5539-0062 has broad-spectrum antibudding activity, we employed the HIV-1 Gag protein in our VLP budding assay and generated HIV-1 Gag-YFP fusion proteins for use in BiMC. Briefly, human 293T cells were cotransfected with NYFP-Tsg101 + CYFP-Gag-WT or CYFP-Gag-ΔP6 (PTAP L-domain mutant) as a control (see Figure 1), and YFP fluorescence was detected at 24 hours p.t. (Figure 5(a)). Cells co-expressing NYFP-Tsg101 + CYFP-Gag-WT displayed a predominantly punctate YFP signal that was evident in both perinuclear patches and at the cell periphery (Figure 5(a)). As expected, the YFP signal was virtually absent in cells co-expressing NYFP-Tsg101 + CYFP-Gag-ΔP6 (Figure 5(a)). Both the CYFP-Gag-WT and CYFP-Gag-ΔP6 fusion proteins were shown to be expressed in transfected 293T cells by western blot (Liu and Harty, data not shown).

 Next, we used both BiMC (Figure 5(b)) and VLP budding (Figure 5(c)) assays to determine whether compound 5539-0062 could inhibit a Gag-Tsg101 interaction and subsequent egress of HIV-1 Gag VLPs in a dose-dependent manner. Human 293T cells were treated with vehicle (DMSO) alone or the indicated concentrations of compound 5539-0062, and then co-transfected with NYFP-Tsg101 + CYFP-Gag-WT (Figure 5(b)). Cells were examined for YFP fluorescence at 24 hours p.t. by confocal microscopy (Figure 5(b)). Abundant YFP positive cells were observed in the presence of DMSO; however, the total number of YFP positive cells decreased with increasing concentrations of compound 5539-0062 (Figure 5(b)). These findings indicate that compound 5539-0062 was able to inhibit the PTAP-mediated interaction between Tsg101 and HIV-1 Gag in a dose-dependent manner. 

 Next, human 293T cells were first treated for 1 hour with either vehicle (DMSO) alone, or increasing concentrations of compound 5539-0062, and then transfected with HIV-1 p55-Gag expression plasmid (Figure 5(c)). p55-Gag was detected by western blot and quantified in both VLPs and cell extracts at 24 hp.t. (Figure 5(c)). Budding of p55-Gag was inhibited by compound 5539-0062 in a dose-dependent manner as shown by decreasing levels of p55-Gag in VLPs (Figure 5(c)). Equivalent levels of p55-Gag were maintained in cell extracts over the entire range of inhibitor concentrations (Figure 5(c)). Taken together, these data indicate that L-domain inhibitors such as compound 5539-0062, are likely to possess broad-spectrum antiviral activity against a wide array of RNA viruses that depend on specific L-domain/host interactions for efficient egress and spread.

## 4. Discussion

 Filovirus-host interactions are important for efficient egress of virus particles; however, mechanistic details of the formation, dynamics, and trafficking of these virus-host complexes in the natural environment of the host cell have been elusive. In this report, we used a BiMC approach to visualize eVP40-Tsg101 complexes as they formed in the cell. The specificity of this interaction was confirmed by using an L-domain deletion mutant of eVP40 and by using Tsg101 specific siRNAs. Importantly, the NYFP-Tsg101 fusion protein was stably expressed in mammalian cells, and CYFP-VP40 fusion proteins retained their ability to bud independently from cells as VLPs in an L-domain-dependent manner. The BiMC approach is ideal for detecting weak and/or transient protein-protein interactions in living cells [27, 28]. We demonstrated that eVP40-Tsg101 complexes formed between 3–4 hours after transfection and colocalized at early times (6 hrs. p.t.) with pericentrin-B, an MTOC marker. We postulate that the initial eVP40-Tsg101 complexes may then migrate from the MTOC to the site of budding at the plasma membrane by 12–24 hours p.t. This working model correlates with previous reports which suggested that filovirus VP40 proteins may interact with and utilize the host cytoskeletal network during assembly and egress [43, 44].

 Elucidation of the molecular complexities and dynamics of virus-host interactions in the natural cell environment will enhance our ability to effectively screen and validate new antivirals [29, 31, 33]. Recent studies have identified two compounds, FGI-104 and FGI-106, that showed activity against filoviruses in cell culture and in animals [45, 46]; however, the targets of FGI-104 and FGI-106 remain to be determined. In addition, small molecule inhibitors of filovirus entry have recently been identified and characterized [47–49]. Viral L-domain/host interactions remain an attractive target for the development of novel, broad-spectrum budding inhibitors [33–35, 50]. The successful development of the BiMC assay to assess filovirus-host interactions (this manuscript) and the use of our well-established VLP budding assay represent powerful tools that will allow us to screen and validate small molecule inhibitors of filovirus budding. By using the known 3D atomic structure of Tsg101 binding to the PTAP motif, we employed an *in silico* strategy to identify and rank commercially available compounds with predicted drug-like properties that could potentially block this interaction. From this screen, we identified compound 5539-0062 and used both BiMC and VLP budding assays to demonstrate that this small molecule specifically inhibited PTAP-dependent budding of eVP40 VLPs, but not PTAP-independent budding of mVP40 VLPs. Moreover, compound 5539-0062 also exhibited specific antiviral activity in cells infected with a recombinant VSV (VSV-M40) expressing the PTAP L-domain of eVP40. The reason for the low level of inhibition of PPxY-dependent budding of VSV-WT observed in the presence of compound 5539-0062 remains to be determined (Table 1), as does the potential effect of compound 5539-0062 on other stages of VSV replication. The PTAP-specific inhibitory activity exhibited by this single, first-generation compound is encouraging and serves as proof-of-principle for using this strategy and the BiMC assay to identify and validate budding inhibitors. Compound 5539-0062 could potentially be optimized to improve binding affinity by either searching for chemically similar molecules in the commercially available databases, or by using the structural interaction fingerprint method which involves selecting compounds which are predicted to have the same protein-ligand interactions as 5539-0062 [51]. One advantage of L-domain inhibitors is their potential broad-spectrum activity against a wide-array of RNA viruses. Indeed, we were able to show that in addition to inhibiting budding of eVP40 VLPs, compound 5539-0062 was also able to block the PTAP-mediated interaction between Tsg101 and HIV-1 Gag, leading to inhibition of HIV-1 Gag budding. 

 Although the focus here was on inhibitors of PTAP-type L-domain activity, similar studies are underway to identify inhibitors of PPxY-type L-domains as well (Liu, Lee, Olson, and Harty, unpublished data). Viral PPxY-type L-domains are known to interact with host proteins such as Nedd4 E3 ubiquitin ligase [4, 24]. One could envision that the use of a cocktail of budding inhibitors containing both PTAP- and PPxY-specific compounds, for example, might be more effective at blocking virus egress than single L-domain inhibitors. Indeed, both EBOV and MARV appear to utilize both PTAP and PPxY L-domains for efficient egress [6, 12]. 

 In addition to these virus-host interaction studies, we are investigating the contributions of viral VP40-VP40, VP40-NP, and VP40-VP35 interactions to filovirus assembly and egress [5, 7, 32, 52]. The broad applicability of the BiMC approach as well as a multicolor fluorescence strategy will enhance our understanding of the biological relevance of these complex multiprotein interactions to RNA virus egress. Extension of these studies to include live virus and/or animal models is essential and will help lead to new insights into filovirus pathogenesis and novel treatment options.

## Figures and Tables

**Table 1:** Viral titers in the presence of drug 5539-0062.

5539-0062 (*μ*M)	VSV-WT		VSV-M40	
	Exp. number 1	Exp. number 2	Exp. number 1	Exp. number 2
0.0	1.2 × 10^5^	1.2 × 10^5^	1.2 × 10^5^	1.1 × 10^5^
25.0	8.2 × 10^4^	8.0 × 10^4^	1.4 × 10^4^	1.0 × 10^4^
50.0	1.5 × 10^4^	1.6 × 10^4^	3.6 × 10^3^	3.8 × 10^3^
75.0	8.6 × 10^3^	N.D.	6.4 × 10^2^	N.D.

N.D. = not done.
